# Old Times

**Published:** 1870-07

**Authors:** 


### OLD TIMES.

We were happy then, but have just found it out. Happy, when we used to go down into the kitchen and "sop" out of the skillet with the little niggers about our own size,—Joe, and Bill, and Tom, and Jenny.

It was a great delight to be permitted to go barefoot, and pull up our trowserloons and wade in the gutters, just after a rain.

We have no such bliss now, as at the moment the first kite we ever made rose gracefully into the upper air; the first time we ever wore a coat with a tail to it; the first time we sported a breastpin; the first pair of kid gloves, the first cravat, the first pair of boots, the first gold watch, the first time we loved, the first time we walked on the street with the eldest born toddling by our side, holding on to the forefinger, with its little, soft, fat, warm hand!

When the two, or three, or four, or half a dozen little ones were gathered about us, with their noises, their mischiefs, their wrangles, their teasings, and their innumerable wants, we now feel as if these were days of sunshine to us; what would we not give to be back there again! Golconda's mines could not purchase that pleasure now; our sons have grown up, have gone out into the world, and have become business men; our daughters are married, and are absorbed in their own sunshine; and some, it may be, rest in the grave; the sparkling eye, the merry laugh, the singing voice, the roguish look, the trusting, loving hearts, all now forever still, silent, dead. Let those who can, enjoy life's gladness as it flies, and drink it in to the full; thus waking up life's energies, imparting new vigor to the circulation, and making us young at fourscore.

